# The Use of Probiotic Strains in Caries Prevention: A Systematic Review

**DOI:** 10.3390/nu5072530

**Published:** 2013-07-05

**Authors:** Maria Grazia Cagetti, Stefano Mastroberardino, Egle Milia, Fabio Cocco, Peter Lingström, Guglielmo Campus

**Affiliations:** 1 WHO Collaborating Centre of Milan for Epidemiology and Community Dentistry, Department of Health Sciences, University of Milan, Milan 20142, Italy; E-Mails: maria.cagetti@unimi.it (M.G.C.); stefano.mastroberardino@hotmail.it (S.M.); 2 Department of Surgery, Microsurgery and Medical Sciences, School of Dentistry, University of Sassari, Sassari 07100, Italy; E-Mail: emilia@uniss.it; 3 Department of Chemistry and Pharmacy, University of Sassari, Sassari 07100, Italy; E-Mail: fcocco@uniss.it; 4 Department of Cariology, Institute of Odontology, The Sahlgrenska Academy, University of Gothenburg, Gothenburg SE-405 30, Sweden; E-Mail: peter.lingstrom@odontologi.gu.se

**Keywords:** probiotics, dental caries, dental caries prevention, cariogenic bacteria, mutans streptococci, lactobacilli, plaque pH, plaque acidogenicity

## Abstract

This paper aims to provide a systematic review of the caries-prevention effect of probiotics in human. The hypothesis was that the administration of probiotic strains might play a role in caries lesion prevention and in the control of caries-related risk factors. The main relevant databases (Medline, Embase) were searched. Quality of the Randomized Clinical Trials (RCTs) was classified using the “Consolidated Standards of Reporting Trials” (CONSORT) checklist and the Impact Factor (IF) value of each journal was recorded. Sixty-six papers were identified, and 23 fulfilled the inclusion criteria. Only three studies had caries lesion development as outcome, all the others reported caries risk factors as interim evaluation. Using the CONSORT Score, the papers were coded as 4 excellent, 9 good and 10 poor. The mean IF value recorded was 1.438. Probiotics may play a role as antagonistic agent on mutans streptococci (MS), acidogenic/aciduric bacteria that contributes to the caries process. In two-thirds of the selected papers, probiotics have demonstrated the capacity to reduce MS counts in saliva and/or plaque in short-term. The effect of probiotics on the development of caries lesion seems encouraging, but to date, RCTs on this topic are insufficient to provide scientific clinical evidence.

## 1. Introduction

Dental caries still remains one of the most common diseases worldwide, although a decline of the prevalence has been recorded in western countries [1,2,3]. The disease is triggered by the interaction over time among cariogenic microorganisms (mainly mutans streptococci and lactobacilli), a diet rich in fermentable carbohydrates and host factors, like as saliva secretion rate and buffering capacity [4]. Mutans streptococci (MS) have been considered for a long time the major pathogens involved in caries development. Nevertheless, in recent years, it was described that the microflora on the tooth surface changes with caries lesion development, from a predominance of non-mutans streptococci and *Actinomyces* spp. to dominance of MS and other non-mutans bacteria, including lactobacilli and *Bifidobacterium* spp. [5].

When sugared food/drinks are supplied frequently, acidogenic and aciduric strains increase selectively in the oral environment. These changes, over time, shift the demineralization/remineralization balance toward net mineral loss, leading to the caries lesion development [6]. Preventive strategies are needed and recommended to control caries risk factors mainly based on dietary changes *i.e.*, sweeteners intake reduction and enhancing host resistance [7,8]. Sometimes, antibacterial agents are administered in order to reduce cariogenic micro-flora, however, a complete eradication of caries-associated microorganisms has proved to be difficult and almost impossible to obtain [9].

The World Health Organization has defined probiotics as “Live microorganisms which, when administered in adequate amounts, confer a health benefit on the host” [10]. These microorganisms belong to the natural human flora in order to survive in the acid environment during transit to the intestines.

Probiotics are recognized to perform several actions in the digestive system as to prevent cellular adhesion and invasion of pathogenic bacteria, modify the intestinal environment and modulating the local and systemic inflammatory immune response [11].

Recent reviews have reported on the use of probiotic strains for the prevention of oral diseases, including caries [12,13]. Probiotics are administered to maintain or restore the natural saprophytic micro-flora against a pathogen invasion, which is central to the development of the major oral diseases (caries and periodontal disease). Probiotic strains administered for oral care are microorganisms mainly used to obtain gastrointestinal benefits, so they might not be ideal for the oral environment, quite different from the intestinal habitat. The effect of probiotics on dental caries and its related risk factors has been evaluated in several experimental studies [14,15,16,17,18,19,20,21,22,23,24,25,26,27,28,29,30,31,32,33,34,35,36], using different strains; *Lactobacillus rhamnosus* GG, *L. casei*, *L. reuteri*, *L. plantarum*, *L. brevis* CD2, *Bifidobacterium* spp. *etc.* were proposed and used to obtain caries incidence reduction, mutans streptococci and lactobacilli count change, plaque pH control and root caries lesions reversal.

Several appropriate vehicles of administration of probiotic strains have been proposed. Dairy products supplemented with probiotics are a natural means of oral administration and easily adopted in dietary regime for adults and children. However, specifically formulated devices with slow release of the microbial strain might be needed in order to oral diseases prevention and control.

Another uncertain aspect of the probiotic use is whether the probiotics species really are able to colonize the oral habitat, and how long the microbial shift was induced [37]. It is well established for probiotics in the gastrointestinal tract that they usually colonize for a short time only [38]. Therefore, a prolonged administration of the probiotics bacteria seemed is mandatory to improve the benefits of the treatment.

The hypothesis behind this systematic review was that the administration of probiotic strains might play a role in the caries lesion prevention and in the control of caries-related risk factors.

## 2. Materials and Methods

### 2.1. Eligibility Criteria

The studies included in the present review are Randomized Clinical Trials assessing the *in vivo* role of probiotics administration on caries lesion development and on caries risk factors control (cariogenic micro-flora, plaque pH, *etc.*). Only human studies considering subjects without any stated medical condition were considered. Only studies in English were collected, due to the virtual absence of research published in other languages as a result of preliminary electronic database searches. All *in vitro* studies, all studies not focusing on probiotics administration for caries prevention and studies where probiotics were administered for other reasons were excluded.

### 2.2. Search Strategy

The main important electronic databases were searched: Medline from 01 January 1966 to 15 May 2013 and Embase from 1973 to 15 May 2013. Two preliminary searches were conducted in March 2013 in order to obtain an overall idea of findings and to polish search terms (MeSH words) and limits. The MeSH Browser was accessed to identify entry terms and compose the final Boolean searches [39].

The first step was the association of MeSH terms Dental Caries and Probiotic(s); after that, a combination of key words derived from the two previous MeSH terms were searched for a total of 18 inquiries. The key words used were: Caries, Probiotic Bacteria, Probiotic Lactobacilli, Bacteriotherapy, Dental Disease, Oral Health, Oral Streptococci, Cariogenic bacteria, Plaque pH and Dental Caries Susceptibility.

A comparison of the 18 different searches was carried out to delete the repeated studies. Then, two authors (M.G.C. and S.M.) examined independently all abstracts of the selected papers. All studies, which appeared to meet the inclusion criteria, were obtained in the full text format. The two authors assessed the papers independently, to establish whether or not the studies met the inclusion criteria.

Disagreements were resolved by discussion. If not possible, other authors were consulted. All studies meeting the inclusion criteria then went to a validity assessment. Studies rejected at this or subsequent stages are reported in the Table 1 of excluded studies with the reasons for exclusion [40,41,42,43,44,45,46,47,48,49,50,51,52,53,54,55,56,57,58,59,60,61,62,63,64,65,66,67,68,69,70,71,72,73,74,75,76,77,78,79,80,81,82]. For each trial, the following information was recorded: citation details; participants: including demographic characteristics and criteria for inclusion; intervention: including type and duration of intervention, duration of follow-up and method of administration.

**Table 1:** List of papers not included in the review.

List of excluded studies	Reasons for exclusion (all different outcome)
Keller *et al.* [40]	Oral malodour
Wang *et al.* [41]	Intestinal health
Allen *et al.* [42]	Diarrhoea
Iniesta *et al.* [43]	Gingival health
Slawik *et al.* [44]	Gingival health
Vandenplas *et al.* [45]	Acute gastroenteritis
Burton *et al.* [46]	Safety and tolerance
Krauss-Silva *et al.* [47]	Preterm delivery
Hummelen *et al.* [48]	Human immunodeficiency virus (HIV)
Harini *et al.* [49]	Gingival health
Saxelin *et al.* [50]	Gastrointestinal persistence
Hummelen *et al.* [51]	Bacterial vaginosis
Arroyo *et al.* [52]	Infectious mastitis
Grossi *et al.* [53]	Diarrhoea
Sierra *et al.* [54]	Intestinal effect
Sinkiewicz *et al.* [55]	Gingival health
Mayanagi *et al.* [56]	Gingival health
Dommels *et al.* [57]	Intestinal persistence
Ranganathan *et al.* [58]	Kidney disease
Twetman *et al.* [59]	Gingival health
Basu *et al.* [60]	Diarrhoea
Staab *et al.* [61]	Gingival health
Mao *et al.* [62]	Diarrhoea
Shimauchi *et al.* [63]	Gingival health
Marcone *et al.* [64]	Bacterial vaginosis
Panigrahi *et al.* [65]	Neonatal gut colonization
Mohan *et al.* [66]	Intestinal health
Ivory *et al.* [67]	Allergic rhinitis
Htwe *et al.* [68]	Diarrhoea
Larsson *et al.* [69]	Bacterial vaginosis
Hatakka *et al.* [70]	Oral candida
Basu *et al.* [71]	Diarrhoea
Henker *et al.* [72]	Diarrhoea
Sugawara *et al.* [73]	Biliary cancer surgery
Krasse *et al.* [74]	Gingival health
Margreiter *et al.* [75]	Diarrhoea
Olivares *et al.* [76]	Intestinal health
Sarker *et al.* [77]	Diarrhoea
Schrezenmeir *et al.* [78]	Acute bacterial infections
Reid *et al.* [79]	Bacterial vaginosis
Morelli *et al.* [80]	Vaginal colonization
Reid *et al.* [81]	Vaginal colonization
Arvola *et al.* [82]	Diarrhoea

### 2.3. Quality Assessment

The quality of the trials was assessed through the “Consolidated Standards of Reporting Trials” (CONSORT) guidelines [83], using the CONSORT 2010 checklist. The 25-items checklist is focused on how the trial was designed, analyzed and interpreted. The quality was classified in three categories according to CONSORT score: excellent (≥20 items), good (between 13 and 19 items) and poor (≤12 items) [84].

The Impact Factor, for each journal where the RCTs were published, was determined from ISI Journal Citation Report, 2011 JCR Science Edition [85].

## 3. Results

Sixty-six (66) papers were identified and assessed, and of these, 23 fulfilled the inclusion criteria and they are reported in Table 2, Table 3, Table 4 [14,15,16,17,18,19,20,21,22,23,24,25,26,27,28,29,30,31,32,33,34,35,36].

No differences were observed between the two main databases used. Selected papers were divided between those performed on children/adolescents and those on adults. All studies utilized parallel arms with intervention and a placebo/control or a crossover design. The sample sizes were generally small or medium, and the majority of them (80%) were short-term interventions (between 10 and 42 days). Different vehicles for the administration and different dosage of probiotics were used. The quality of published papers recorded using the Consort Score was: 4 excellent, 9 good and 10 poor. All papers, except two [19,23], were published on Journals with positive IF with a mean value of 1.438.

**Table 2:** Studies with caries risk factors as outcome (children/adolescents).

Reference Study design	Outcome(s)	Subjects Age	Strain (Concentration)	Delivery System/Treatment Duration	Groups	Results	Consort score	IF score
Taipale *et al.*, 2013 [14]	MS in plaque (plate culturing)	106 children (4 years)	*Bifidobacterium animalis* subsp. *lactis* BB-12 (10^1^^0^ CFU/mL)	Tablets in slow-release pacifier or spoon twice daily/22–23 months	A: Probiotic B: Xylitol C: Sorbitol	No statistically significant MS differences among groups	excellent	2.328
Campus *et al.*, 2013 [15]	MS in saliva and plaque pH (plate culturing)	191 children (6–8 years)	*Lactobacillus brevis* CD2 (2 × 10^9^/g)	Lozenges twice a day/6 weeks	A: Probiotic B: Placebo	Statistically significant decrease in MS and increase in plaque pH in group A	excellent	2.364
Juneja *et al.*, 2012 [16]	MS in saliva (chair-side tests)	40 children (12–15 years)	*Lactobacillus rhamnosus* hct 70 (2.34 × 10^9^ CFU/day)	Milk twice daily/3 weeks	A: Milk B: Milk + Probiotic	Statistically significant reduction in MS immediately after consumption and after 3 week follow-up in group A	poor	0.444
Taipale *et al.*, 2012 [17]	MS in plaque and Lb and yeasts in mucosa/teeth (plate culturing)	106 infants (1 month)	*Bifidobacterium animalis* subsp. *lactis* BB-12 (10^1^^0^ CFU/mL)	Tablets in slow-release pacifier or spoon twice daily/months	A: Probiotic B: Xylitol C: Sorbitol	MS colonization statistically significant differ, lactobacilli and yeasts not differ among groups	excellent	2.328
Singh *et al.*, 2011 [21] cross-over study	MS and Lb in saliva (chair-side tests)	40 children (12–14 years)	*Bifidobacterium lactis* Bb-12 ATCC27536 and *Lactobacillus acidophilus* La-5 (10^6^ CFU/g)	Ice-cream/10 days	A: Ice-cream B: Ice-cream/probiotics	Statistically significant reduction in MS in group B, but no significant effect on lactobacilli	good	1.066
Aminabadi *et al.*, 2011 [22]	MS in saliva (plate culturing)	105 children (6–12 years)	*Lactobacillus rhamnosus* GG (2 × 10^8^ CFU/mL)	Yogurt/3 weeks (chlorhexidine mouthrinse 2 weeks)	A: Chlorhexidine B: Probiotic C: Chlorhexidine, than probiotic	Statistically significant MS decrease immediately after probiotic use in group B; recolonization during the 5 consecutive weeks. In group C a statistically significant MS reduction that enhances during the 5 consecutive weeks	good	2.328
Jindal *et al.*, 2011 [23]	MS in saliva (plate culturing)	150 children (7–14 years)	*Lactobacillus rhamnosus*, *Bifidobacterium longum*, *Saccharomyces cereviasae* (1.25 billion) *Bacillus coagulans* (150 million)	Powders (dissolved in water and used as mouthrinse)/14 days	A: Placebo B: *L. rhamnosus, B. longum* and *S. cereviasae* C: *B. coagulans*	Statistically significant MS reduction in groups B and C	good	-
Lexner *et al.*, 2010 [26]	MS and Lb in saliva (plate culturing)	18 adolescents (13–17 years)	*Lactobacillus rhamnosus* LB21 (10^7^ CFU/mL)	Milk once daily/2 weeks	A: Probiotic B: Placebo	No statistically significant MS reduction and Lb	poor	0.539
Cildir *et al.*, 2009 [27] cross-over study	MS and Lb in saliva (chair-side tests)	24 adolescents with fixed orthodontics (12–16 years)	*Bifidobacterium animalis* subsp. *lactis* DN 173010 (2 × 10^8^ CFU/g)	Yogurt once daily/2 weeks	A: Probiotic B: Placebo	Statistically significant MS reduction in group A and no significant Lb alterations	poor	0.975
Stecksén-Blicks *et al.*, 2009 [28]	MS and Lb in plaque (plate culturing)	248 children (1–4 years)	*Lactobacillus rhamnosus* LB21 (10^7^ CFU/mL)	Milk/21 months	A: Probiotic/fluoride B: Placebo	No statistically significant changes in MS and Lb	good	2.462
Näse *et al.*, 2001 [36]	MS in plaque and saliva (chair-side tests)	594 children (1–6 years)	*Lactobacillus rhamnosus* GG, ATCC 53103 (5–10 × 10^5^ CFU/mL)	Milk five daily/7 months	A: Milk/probiotic B: Milk	Statistically significant MS reduction in group A	excellent	1.667

**Table 3:** Studies with caries risk factors as outcome (adults).

Reference Study design	Outcome(s)	Subjects Age	Strain (Concentration)	Delivery System/Treatment Duration	Groups	Results	Consort score	IF score
Marttinen *et al.*, 2012 [18] Cross-over study	Plaque acidogenicity, MS and Lb in plaque (plate culturing)	13 adults (mean 25 years)	*Lactobacillus rhamnosus* GG or *Lactobacillus reuteri* (196 million CFU/tablet)	Tablet twice a day/2 weeks	A: LGG B: *L. reuteri*	No changes in plaque acidogenicity. MS remained stable, while Lb increased in the *L. reuteri* group, but not in the LGG group	good	2.364
Keller & Twetman, 2012 [19] Cross-over study	MS and Lb in saliva (chair-side tests) Lactatic Acid production in plaque	18 adults (mean 26 years)	*Lactobacillus reuteri* (DSM 17938 and ATCC PTA 5289) (2 × 10^8^ CFU/tablet)	Tablets three times a day/2 weeks	A: *L. reuteri* B: Placebo	No statistically significant MS change; Lb increased significantly in group A. No significant differences in Lactatic Acid production	good	-
Keller *et al.*, 2012 [20]	Inhibiting regrowth of salivary MS after full-mouth disinfection (chair-side tests)	62 adults (mean 23 years)	*Lactobacillus reuteri* (DSM 17938 and ATCC PTA 5289) (2 × 10^8^ CFU/tablet)	Tablets twice daily/6 weeks	A: Probiotics B: Placebo	*L. reuteri* did not seem to affect or delay the regrowth of MS	good	2.328
Petersson *et al.*, 2011 [24]	MS and Lb in saliva (chair-side tests) and plaque (plate culturing)	160 adults (58–84 years)	*Lactobacillus rhamnosus* LB21 (10^7^ CFU/mL)	Milk once daily/15 months	A: Placebo B: Fluoride/probiotic C: Probiotic D: Fluoride	Lower prevalence of MS and Lb, but not statistically significant	good	1.066
Chuang *et al.*, 2011 [25]	MS and Lb in saliva (chair-side tests) and buffer capacity (Dentobuff strip)	80 adults (20–26 years)	*Lactobacillus paracasei* GMNL-33 (3 × 10^8^ CFU/mL)	Tablets three times per day/2 weeks	A: Probiotics B: Xylitol	No statistically significant differences in MS and Lb and buffer capacity. MS reduction intra probiotics group	poor	2.364
Caglar. *et al.*, 2008 [29] Cross-over study	MS and Lb in saliva (chair-side tests)	24 adults (mean 20 years)	*Bifidobacterium lactis* Bb-12 (10^7^ CFU/g)	Ice-cream once daily/10 days	A: Probiotic B: Placebo	Statistically significant MS reduction in group A; salivary Lb levels unaltered	poor	1.095
Caglar *et al.*, 2008 [30]	MS and Lb in saliva (chair-side tests)	20 women (mean 20 years)	*Lactobacillus reuteri* ATCC 55730: ATCC PTA 5289 10:1 (1.1 × 10^8^ CFU)	Lozenge once daily/10 days	A: Probiotic B: Placebo	Statistically significant MS reduction in group A; Lb unaltered	poor	1.072
Caglar *et al.*, 2007 [31]	MS and Lb in saliva (chair-side tests)	80 adults (21–24 years)	*Lactobacilli reuteri* ATCC and *Lactobacilli reuteri* ATCC PTA 5289 (10^8^ CFU/gum)	chewing gums three times daily/3 weeks	A: Probiotics B: Xylitol C: Probiotics/xylitol D: Placebo	Statistically significant MS reduction in group A, B and C; Probiotic + xylitol not enhance the efficacy.	poor	1.956
Caglar *et al.*, 2006 [32]	MS and Lb in saliva (chair-side tests)	120 adults (21–24 years)	*Lactobacillus reuteri* ATCC 55730 (10^8^ CFU/straw or tablet)	Water or tablet once daily/3 weeks	A: Water/probiotic B: Placebo water C: Tablet/probiotic D: Placebo tablet	Statistically significant MS reduction in groups A and C; similar but non-significant trend for Lb	poor	1.017
Caglar *et al.*, 2005 [33] Cross-over study	MS and Lb in saliva (chair-side tests)	26 adults (21–24 years)	*Bifidobacterium* DN-173 010 (7 × 10^7^ CFU/g)	Yogurt once daily/2 weeks	A: Probiotic B: Placebo	Statistically significant MS reduction in group A; similar but non-significant trend for Lb	poor	0.783
Montalto *et al.*, 2004 [34]	MS and Lb in saliva (chair-side tests)	35 adults (23–37 years)	*L. sporogens*, *L. bifidum*, *L. bulgaricus*, *L. termophilus*, *L. acidophilus*, *L. casei*, *L. rhamnosus* (1.88 × 10^9^ live cells/day)	Liquid and capsule/45 days	A: Probiotics capsules placebo in liquid B: Liquid probiotics placebo in capsules C: Placebo in both liquid and capsule	Statistically significant Lb increase in groups A and B. MS not significantly modified.	poor	1.473
Ahola *et al.*, 2002 [35]	MS, Lb and yeasts in saliva (chair-side tests) and buffer capacity (Dentobuff strip)	74 young adults (18–35 years)	*Lactobacillus rhamnosus* GG ATCC 53103 (1.9 × 10^7^ CFU/g) and *Lactobacillus rhamnosus* LC 705 (1.2 × 10^7^ CFU/g)	Cheese five daily/3 weeks	A: Probiotics B: Placebo	No statistically significant differences in MS and Lb after the intervention; during the post-treatment period (3 weeks) a significantly reduction of the two species in group A. No statistically significant differences in yeast and buffer capacity	good	1.047

**Table 4:** Studies with caries lesion development as outcome.

Reference	Outcome(s)	Subjects	Strain (Concentration)	Delivery System/Treatment Duration	Groups	Results	Consort score	IF score
Taipale *et al.*, 2013 [14]	Caries increment (ICDAS index)	106 children (4 years)	*Bifidobacterium animalis* subsp. *lactis* BB-12 (1010 CFU/mL)	Tablets in slow-release pacifier or spoon twice daily/22–23 months	A: Probiotic B: Xylitol C: Sorbitol	No differences in the occurrence of enamel caries	excellent	2.328
Petersson *et al.*, 2011 [24]	Root Caries Index (RCI) and Electric Resistance Measurements (ERM)	160 adults (58–84 years)	*Lactobacillus rhamnosus* LB21 (10^7^ CFU/mL)	Milk once daily/15 months	A: Placebo B: Fluoride/probiotic C: Probiotic D: Fluoride	Higher numbers of RCI reversals in groups B, C and D. Mean ECM values increased significantly in groups A, B and C	good	1.066
Stecksén-Blicks *et al.*, 2009 [28]	Caries increment (dmfs index)	248 children (1–4 years)	*Lactobacillus rhamnosus* LB21 (10^7^ CFU/mL)	Milk once daily/21 months	A: Probiotic/ fluoride B: Placebo	Statistically significant difference in caries increment in group A	good	2.462

### 3.1. Probiotics and Caries Prevention in Children/Adolescents

Eleven studies were evaluated [14,15,16,17,21,22,23,26,27,28,36]. Only one study was performed to verify the effect of the early administration of probiotics (*Bifidobacterium animalis* subsp. *lactis* BB-12) on the oral colonization of mutans streptococci (MS) in 106 infants from a low-caries population [17]. Subjects received probiotic bacteria, xylitol or sorbitol (polyol 100–300 mg) from the age of 1–2 months to the age of 2 years, twice a day. The MS concentration in plaque of the mothers at the start of the study was high and similar in all subjects, without significant differences. At the end of the study, children showed a rather low MS colonization percentage, with a statistically significant difference among groups. At the age of 4 years, the same children were re-evaluated to assess the MS level in plaque and the occurrence of dental caries in deciduous teeth [14]. No differences were observed for both parameters among the three groups.

Otherwise, nine studies were carried out to verify the effect of probiotics strains on MS levels in saliva and/or dental plaque, using different vehicle [15,16,21,22,23,26,27,28,36]. Only two studies did not demonstrate any change in SM level [26,28].

The effect of milk containing *L. rhamnosus* on MS counts was evaluated in four papers (two short and two long-term studies). In the short-term studies [16,26], the effect of milk containing *Lactobacillus rhamnosus* (hct 70 or LB21) for few weeks was registered in small groups of adolescents. The difference in post treatment regarding MS count between test and control group was not statistically significant, while the difference in follow-up was highly significant [16]. No statistically significant differences in SM were recorded in subjects who received milk with probiotic compared to subjects using milk without probiotic [26]. In the long-term studies [28,36], *L. rhamnosus* was administered for several months (7 and 21 months respectively). Statistically significant reductions were recorded with *Lactobacillus rhamnosus* GG, ATCC use [36], while no statistically significant changes were observed in SM counts in subjects receiving *Lactobacillus rhamnosus* LB21 [28].

Two studies were performed with yogurt as probiotics vehicle [22,27]. The effect of the administration of yogurt containing *Lactobacillus rhamnosus* GG for three weeks in 105 children was evaluated with a significant decrease in SM count immediately after probiotics use alone, but recolonization was described during the five consecutive weeks [22]. Pre-treatment with chlorhexidine produced a statistically significant reduction in salivary SM counts that enhances during the five consecutive weeks. A double-blind, crossover study was carried out on 24 healthy adolescents, undergoing orthodontic treatment, with the aim to assess the effect of yogurt containing *Bifidobacterium animalis* subsp. *lactis* DN-173010 administered once daily [27]. Statistically significant reduction of MS was recorded after probiotic yogurt consumption.

One study used ice-cream as probiotic vehicle [21]; a combination of *Bifidobacterium lactis* Bb-12 and *Lactobacillus acidophilus* La-5 was evaluated in 40 adolescents. Significant reduction in salivary MS scores was reported after consumption of the probiotic compared to baseline.

One study was performed using lozenges as probiotic vehicle. The effect of lozenges containing *Lactobacillus brevis* CD2 administered for six weeks was evaluated in 191 high caries risk children [15]. A statistically significant reduction of the cariogenic microorganism was recorded.

One study used two powders as probiotic vehicle in 150 children aged 7–14 years, containing the first *Lactobacillus rhamnosus*, *Bifidobacterium longum* and *Saccharomyces cereviasae* and the second *Bacillus coagulans*, and compared them to a placebo powder [23]. Powders dissolved in 20 mL of water were used as a mouth rinse for one minute for 14 consecutive days. Data analysis showed a statistically significant reduction in MS counts in both probiotics groups.

Five studies of the ten reported above, investigated the effect of the probiotics strain on Lb level also [17,21,26,27,28]. In all studies, a statistically significant change in Lb counts in saliva and/or plaque was not observed. Moreover, one study evaluated the effect of the probiotic on oral yeasts, failing to prove any statistically effect [17]. The effect of probiotics on plaque pH modification after a rinse with a 10% sugared solution was investigated and plaque acidogenicity resulted significantly lower in subjects that have used probiotic lozenges [15]. Two studies evaluated the probiotic effect on caries lesion development [14,28]. A statistically significant difference in caries increment was recorded only in one paper in subjects who received probiotic and fluoride compared to subjects who received placebo milk [28].

### 3.2. Probiotics and Caries Prevention in Adults

Twelve studies were selected [18,19,20,24,25,29,30,31,32,33,34,35]: all of them investigated the effects of probiotic administration on MS counts in plaque and/or saliva and six demonstrated a MS reduction.

Caglar and co-workers [29,30,31,32,33], performed several studies on the change of salivary MS concentration after the use of several probiotics (*Bifidobacterium lactis* Bb-12, *Lactobacillus reuteri* ATCC 55730 and ATCC PTA 5289, *Bifidobacterium* DN-173 010) using different vehicles (ice-cream, chewing-gum, water, yogurt and tablets). MS concentrations decrease significantly in all studies.

No statistically significant differences in MS counts were recorded immediately after consumption of cheese containing *Lactobacillus rhamnosus* GG and *Lactobacillus rhamnosus* LC 705, but a significant reduction was reported three weeks after the experimental period [35].

Conversely, the other six of the twelve studies did not reveal an effect of probiotics administration on MS counts [18,19,20,24,25,34]. Four short-term studies were performed using tablets containing *Lactobacillus rhamnosus* or *Lactobacllus reuteri*; MS counts remained stable after the administration of both probiotics twice a day for two weeks in 13 adults [18]. No significant differences were also observed after the use for two weeks of *Lactobacillus reuteri* on MS counts in 18 adults [19], and using the same strains after full mouth disinfection with chlorhexidine on 62 adults on regrow of MS [20]. Tablets containing *Lactobacillus paracasei* GMNL-33 were unsuccessfully administered to 80 young adults [25]. One long-term study evaluated the effect of *Lactobacillus rhamnosus* LB21 delivered in milk on MS count in saliva and supra-gingival plaque in 160 older adults for 15 months [24]. No statistically significant reduction in MS count was registered. Results from a study utilizing several strains of *Lactobacillus* spp. in liquid and capsules form in 35 adults revealed no significantly MS count reduction [34].

Moreover, ten studies of the twelve reported above, investigated the effect of the probiotics strain on Lb level in saliva and/or plaque [18,19,25,29,30,31,32,33,34,35]. Eight studies failed to prove any effect on Lb counts and two studies demonstrated a statistically significant change in Lb counts [19,35].

Two studies evaluated also the effect of probiotics on plaque acidogenicity, but no significant changes were found [18,19]. Two studies investigated the effect on buffer capacity failing to demonstrate a statistically significant difference on it [25,35]. One of these did not demonstrate an effect on oral yeast yet [35].

## 4. Discussion

The role of the administration of probiotic strains in caries prevention was the aim of this systematic review. Results described by various research groups were encouraging [15,16,17,21,22,23,24,27,29,30,31,32,33,35,36], but the scientific evidence is still unclear and often not very high. The main goal for the use of probiotics in caries prevention is to replace and displace cariogenic bacteria, mainly mutans streptococci, with noncariogenic bacteria [13].

Most clinical trials reviewed had a small sample size and reported caries risk factors as intermediate or surrogate endpoints, which limited the conclusions about the real efficacy of probiotics administration in caries lesion prevention. From the analysis of the RCTs selected, it reasonable to affirm that probiotic strains may play a role as antagonistic agent on cariogenic bacteria. In the two-thirds of the selected papers, probiotics have demonstrated the capacity to reduce MS counts in saliva and/or plaque regardless of the product or strain used. However, this effect is variable and probably short-lasting. In addition, MS are no longer considered the main cariogenic bacteria involved in the caries progress, since the important role of non-mutans acidogenic and aciduric bacteria was clarified [5]. Different results are reported on the effect of probiotics on lactobacilli counts. From the fourteen studies that evaluated the changes of this interim outcome, just two reported a positive result [19,35]. The other interim outcomes considered (yeasts and plaque acidogenicity) were investigated in few studies and the results are unclear. Only three selected papers [14,24,28], two performed on children and another one on adults/elderly samples, had caries lesion development as outcome; two studies reported a statistically significant difference in caries increment after 15/21 months of probiotics use [24,28].

Several mechanisms of action for probiotic are described in literature, same of them still not fully understood. Several local and systemic effects are describing, including adhesion, co-aggregation, competitive inhibition, production of organic acids and bacteriocin-like compounds and immune-modulation [86]. However, probiotic bacteria are not able to colonize oral cavity permanently [29], so a continuous regular, almost daily intake is required. This may be a compliance aspect to be considered.

In eleven selected papers, a dairy product (milk, cheese, yogurt and ice-cream) was used as delivery vehicle for probiotics [16,21,22,24,26,27,28,29,35,36]. These non-sweetened products are known to possess caries preventive effects related to a natural high contents in calcium and phosphate that enhance remineralization of hard oral tissues and contrast acids produced by cariogenic bacteria after sugared foods and drinks intake [13]. Only one selected paper used chewing gums as delivery vehicle [31]. The use of non-sugared chewing gum has been considered useful for dental health, since it reduces plaque acidogenicity and increases enamel remineralization, enhancing salivary flow rate [87]. The remaining eleven papers used as probiotic vehicle products (lozenges, tablets, powders) without any reported preventive effects themselves [14,15,17,18,19,20,23,25,30,32,34].

One study evaluated the combined effect of probiotics and fluoride on cariogenic bacteria and caries lesion increment. No statistically significant differences were recorded between the group using probiotics alone and those using probiotic and fluoride together [24]. Another paper studied the combined effect of probiotics and low dosage of xylitol on cariogenic microorganisms [31], but no statistically significant differences were noted compared to probiotics alone. Finally only one study has investigated the effect of probiotic on MS counts after chlorhexidine mouthwash disinfection [22]. Pre-treatment with chlorhexidine produced a long-lasting reduction in salivary SM compared to probiotics alone.

It is interesting to note that up to day none products have successfully approved by the European Food Safety Authority (EFSA) [88,89].

A theoretical risk of the probiotic assumption is the increase of caries risk due to the capacity of probiotic strains to form biofilm and produce acids, but this aspect was not taken into consideration by any papers.

Two approaches have been used to assess the quality of RCTs in the present review: the CONSORT checklist and the journal Impact Factor. A significant association between the CONSORT score and the impact factor was reported [90].

The CONSORT checklist takes into account 25 important methodological items, providing an accurate evaluation of the methodological correctness with which the study was planned and carried out. From the analysis of the checklists of the selected papers, the main deficiencies observed were the lack of information on methods to define the hypothesis, the sample size calculation, the absence of data on the results of estimated effects size and their precision. These methodological weaknesses reduce the validity of studies and the interpretation of the results may lead to biased findings. Moreover, few studies reported correctly the results of the RCTs not taking into account other sources of bias. In general, the quality of reporting of RCTs was quite low, with half of all studies scoring as poor with the exception of three studies that were scored as excellent. These results are similar to those reported of other systematic review [13], but it is possible to observe a progressive improvement in the scientific evidence of the effect of probiotic on caries prevention.

The journal impact factor has been used widely as a quality measure of the published papers [91]. All selected studies except two were published in journals with impact factor and all except one in dental journal [34]. The mean value of impact factors of the selected studies (1.438) might seem low when compared with IF of journals from other areas of medicine. However, the mean impact factor value of the 81 impacted dental journals is quite low (1.455—range 0.037–3.961), with the journals with the highest IF values dedicated to other topic of dentistry, different from caries prevention. Therefore, the mean IF value recorded in the present review has to be considered a quite good score.

## 5. Conclusions

The use of probiotic strains for caries prevention showed promising results even if only few studies have demonstrated clear clinical outcomes. Therefore, the scientific evidence is still poor. A continuous regular almost daily intake is probably required; this maybe a compliance aspect to be considered. However, for all products effective in caries prevention (*i.e.*, fluoride and chlorhexidine) a frequent intake is required, so a possible way of administration could be to insert probiotic in other daily preventive products like toothpaste.
